# ELEMENTS OF AN INTERPROFESSIONAL LEARNING CULTURE IN NURSING HOMES

**DOI:** 10.1093/geroni/igac059.1518

**Published:** 2022-12-20

**Authors:** Judith Meijers, Merel (E A ) Van Lierop, Frank Verbeek, Miranda Laurant, Erik van Rossum, Sandra Zwakhalen, Anneke Van Vught

**Affiliations:** Maastricht University, Maastricht, Limburg, Netherlands; Maastricht University, Maastricht, Limburg, Netherlands; HAN University, Nijmegen, Gelderland, Netherlands; HAN University, Nijmegen, Gelderland, Netherlands; Zuyd University of Applied Sciences, Maastricht, Limburg, Netherlands; Maastricht University, Maastricht, Limburg, Netherlands; HAN University, Nijmegen, Gelderland, Netherlands

## Abstract

Healthcare professionals in nursing homes need to be able to keep up with changes at work, deal with a growing complexity in healthcare demands and have to keep developing themselves within a team to be able to plan and deliver high quality of care. To keep up with these changes and demands, an interprofessional learning culture must be stimulated. Insight about developing such a culture in nursing homes is lacking. A scoping review was performed. Articles were examined on actions that positively influence the development of an interprofessional learning culture. 51 Actions were identified. The influencing factors were divided in eight main themes i.e. (1) communication, (2) shared goals, (3) tasks and responsibilities, (4) learning and knowledge, (5) work approaches, (6) attitude, (7) leadership, and (8) safety, respect and transparency. These actions form a basis for an interprofessional learning culture but should be operationalized and tailored to practice use.

